# How the brain prevents a second error in a perceptual decision-making task

**DOI:** 10.1038/srep32058

**Published:** 2016-08-18

**Authors:** Rinaldo Livio Perri, Marika Berchicci, Giuliana Lucci, Donatella Spinelli, Francesco Di Russo

**Affiliations:** 1 Department of Movement, Human and Health Sciences, University of Rome “Foro Italico”, Rome, Italy; 2 Department of Psychology, University of Rome “La Sapienza”, Rome, Italy; 3 IRCCS Santa Lucia Foundation, Rome, Italy

## Abstract

In cognitive tasks, error commission is usually followed by a performance characterized by post-error slowing (PES) and post-error improvement of accuracy (PIA). Three theoretical accounts were hypothesized to support these post-error adjustments: the cognitive, the inhibitory, and the orienting account. The aim of the present ERP study was to investigate the neural processes associated with the second error prevention. To this aim, we focused on the preparatory brain activities in a large sample of subjects performing a Go/No-go task. The main results were the enhancement of the prefrontal negativity (pN) component -especially on the right hemisphere- and the reduction of the Bereitschaftspotential (BP) -especially on the left hemisphere- in the post-error trials. The ERP data suggested an increased top-down and inhibitory control, such as the reduced excitability of the premotor areas in the preparation of the trials following error commission. The results were discussed in light of the three theoretical accounts of the post-error adjustments. Additional control analyses supported the view that the adjustments-oriented components (the post-error pN and BP) are separated by the error-related potentials (Ne and Pe), even if all these activities represent a cascade of processes triggered by error-commission.

The ancient Romans used to say that *“tempus omnia medetur”* (i.e., time heals all); however, time is not always unlimited and sometimes it is important to immediately adjust behavior to avoid a new error. Understanding how the brain prevents its own errors is a current challenge for neuroscientists. In healthy people, error commission in tasks such as Go/No-go or stop signal is usually followed by conscious experience of incorrect response, allowed by a neural system specialized in error detection [see, e.g. ref. 1]. This system has been largely investigated by means of event-related potential (ERP) studies^2^,^3^,^4^, describing the error-related negativity (ERN) or error negativity (Ne, peaking at 50–100 ms after the error), and the error positivity (Pe, peaking at 200–300 ms after the error)^5^,^6^,^7^. Functional magnetic resonance imaging (fMRI) studies in this topic [for a review see ref. 8] showed the frontal and parietal brain regions activation immediately following error commission. In contrast, less is known about brain activity that prepares and adjusts the immediate action subsequent to an error. Studying this stage of processing is the main goal of the present study.

At a behavioral level, error commission leads to post-error adjustments whose effects typically emerge through *post-error improvement of accuracy* (PIA) [e.g. ref. 9] and *post-error slowing* (PES) [e.g. ref. 10], reflecting increased accuracy and slower response times (RTs) in post-error trials, respectively. According to Danielmeier and Ullsperger’s review^11^, three accounts may explain the PES. (1) The *cognitive control* account describes the PES as the result of increased top-down control, as revealed by the relationship between the activity of the medial frontal cortex (MFC) and behavioral slowing [e.g. ref. 12]. Some studies also underlined the association between the MFC and adjustments at the level of the response priming unit^13^: in other words, the reduced activity in motor areas would predict post-error slowing^14^,^15^. (2) The *inhibitory account* states that PES is supported by increased inhibition in trials following error commission^16^. The inhibition is sustained by the activation of the right PFC^9^ and plays a central role in motor slowing, because the PFC is part of the proactive inhibitory network of the right hemisphere [e.g. ref. 17]. (3) According to the *orienting account*, PES emerges after any kind of infrequent event. In other words, response slowing may also occur after correct responses (i.e., post-correct slowing)^18^ if these latter are infrequent.

Studies conducted with fMRI shed light on the neural substrates of post-error adjustments, especially on the engagement of the PFC and premotor regions in the behavioral slowdown^12^,^14^,^19^; however, due to the low temporal resolution, this technique does not always allow to distinguish between the error-detection processing and the following adjustment mechanisms^19^. On the other hand, several electroencephalographic (EEG) studies attempted to investigate the PES using approaches based on both frequency and ERP analysis. For example, Cavanagh and colleagues^20^ reported increased theta oscillation at mid-frontal and lateral frontal sites immediately after the error. Further, they showed a relationship between theta band and behavioral adjustments. However, since theta oscillations are supposed to reflect the error-detection process^21^, we can hypothesize that the relationship between theta power and post-error adjustments is not direct, but could be mediated by an additional processing occurring later than that described by Cavanagh and colleagues^20^. This hypothesis is supported by the inconsistency among ERP results: some studies reported an association between PES and Ne^6^,^22^,^23^,^24^, while others found a correlation between PES and Pe^4^,^25^,^26^. These contradictory findings could be partly explained by the results of Marco-Pallarés and colleagues^9^, who reported an association between the increased frontal-central beta activity at 600–800 ms after the error (i.e., 400 ms after the Pe) and the behavioral slowdown, suggesting that motor inhibition processes (as reflected by beta increase^27^) occur after the error and may account for the PES. It is noteworthy that the neurocognitive dissociation between conscious detection of error and the adjustment-oriented processes was also supported by a study on cocaine users that showed reduced awareness of errors, but intact performance adjustments^28^.

In sum, ERP studies that investigated the post-error adjustments reported an association between the post-error slowing and one of the two error-related potentials (the Ne and Pe). However, any decision-making behavior is “primed” by preparatory activities that have a direct relationship with the motor response [e.g. ref. 29]. Accordingly, our hypothesis is that error commission leads to neural adjustment mechanisms that regulate the post-error performance, and these adjustments should be the focus of investigation rather than error-detection activities. To the best of our knowledge, this is the first ERP study investigating the post-error *neural* adjustments, that is the stage of processing following the error awareness stage (the Pe component) and preceding the post-error response. To this aim, we focused on the preparation processing taking place long before action, and we considered both cognitive preparation (as indexed by the prefrontal negativity or pN^30^) and motor preparation activities (as indexed by the Bereitschaftspotential or BP^31^). Specifically, we compared the preparatory brain activity of trials following errors (hereafter post-error trials) with those following correct responses (hereafter post-correct trials) in an equiprobable Go/No-go task. The advantage of the equiprobable task is to exclude confounding factors such as the strong response tendency (typical of the tasks with high frequency of Go trials). The disadvantage is that the number of errors is low when compared to higher frequency of Go trials; however, the 50/50 ratio allows a more reliable comparison between Go and No-go conditions.

It is noteworthy that the ERP investigation of post-error adjustment mechanisms is possible only if error-related potentials (Ne and Pe) do not overlap with preparatory activities of post-error trials. To verify this prerequisite, we performed control analyses confirming that the present paradigm does allow isolating two sets of consecutive processing: the first, related to the detection of the previous error, the second, to the preparation of the current trial.

According to the cognitive account of the PES^12^, we would expect an increased top-down control during the preparation of post-error trials, which is associated with increased activity of frontal-medial regions. At the ERP level, the increased top-down control should emerge through bilateral enhancement of the pN component^32^,^33^. Further, since it was also proposed that the activation of the MPFC predicts the reduced premotor activity^15^, we may expect an amplitude reduction of the BP component in post-error trials. In fact, the BP mainly reflects the activity of the supplementary motor area (SMA^31^) and its amplitude was associated with the motor baseline modulating the response speed^29^,^30^. According to the inhibitory account (as reviewed by^11^), we may expect a selective right-side enhancement of the pN: in fact, this component was localized in the inferior frontal gyrus (iFg; ref. 34), which plays a key-role in the proactive inhibitory control^17^,^35^,^36^. Because of the lack of conditions modulating the frequency of errors (or correct responses), the present study is not suited to test directly the orienting account. However, since the errors represent infrequent events in this paradigm, the present results can also be interpreted in terms of the orienting account.

Finally, since previous studies found a positive relationship between the amplitude of the N1 and P1 potentials, and the speed and accuracy performance respectively^30^,^37^, we also investigated whether the post-error behavior might be partially mediated at the visual processing level.

## Material and Methods

### Ethics statement

This project, including the informed consent, and all experimental protocols, was approved by the Ethics Committee of the Santa Lucia Foundation (CE-AG-4/56). All methods were carried out in accordance with the approved guidelines and regulations. Informed consent was obtained from all subjects.

### Subjects

From a database of 108 subjects who participated in the Go/No-go task (described below), we excluded 11 subjects that did not report false alarms (FAs, i.e. responses to No-go stimuli) and processed the electroencephalographic (EEG) data of the remaining subjects (n = 97). Among those making FAs, 56 participants (corresponding to the 57% of the sample) reported more than 20 FAs, but only those with at least 20 artifact-free FAs were considered for the grand-averages. By this procedure, 36 subjects were selected for the final group (6 females; mean age = 38.9, SD = 11.3): the mean percentage of FAs was 11.1%, SD = 6.2 (range: 3–27.7).

The participants had normal or corrected-to-normal vision and no history of neurological or psychiatric disorders; all subjects were right-handed (Edinburgh handedness inventory^38^).

### Procedure and Task

**Figure 1:**
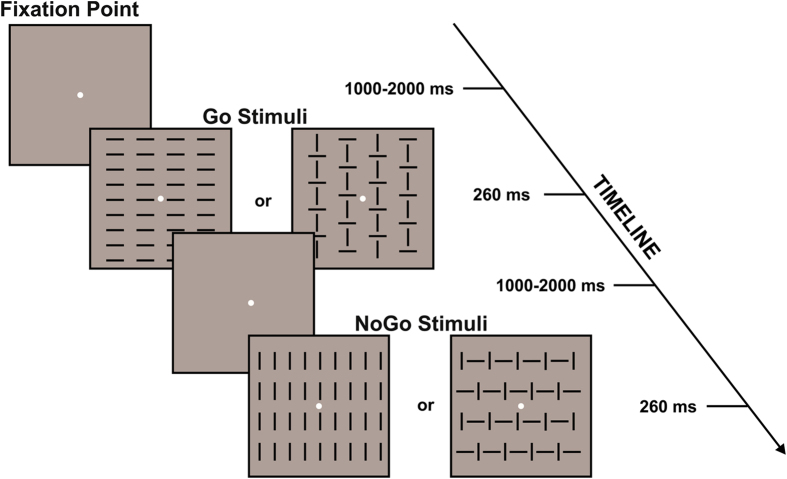
Schematic representation of the stimuli and paradigm adopted in the present Go/No-go task.

Subjects were tested in a sound attenuated, dimly lit room; they were comfortably seated in front of a computer monitor at a distance of 114 cm, and a board was fixed on the armchair allowing them to freely push the button panel positioned on it. A yellow circle (subtending 0.15° × 0.15° visual angle) constantly displayed at the center of the screen served as the fixation point. The four visual stimuli consisted of squared configurations (subtending 4° × 4°) made of vertical or horizontal segments, or both of them with different orientation (vertical and horizontal) presented centrally on a dark gray background. Two configurations were defined as targets (Go stimuli) and two as non-targets (No-go stimuli). The four stimuli were randomly presented for 260 ms with equal probability (p = 0.25). The stimulus-onset asynchrony varied from 1000 to 2000 ms in order to avoid time prediction effects and confounding overlapping activity from previous and following trials. See Fig. 1 for a detailed representation of stimuli and procedure. The experiment consisted of a total of 10 blocks, each of which contained 80 trials and lasted 2.5 min with an inter-trial rest period. A total of 800 trials were delivered in the experiment: 400 for Go and 400 for No-go category. The total duration was about 30 min, depending on the subjective rest time.

Participants were asked to press a button with the right index finger when Go stimuli appeared on the monitor, and withhold the response when No-Go stimuli appeared, and to be as accurate and fast as possible.

### Behavioral recording and analysis

We calculated the error percentage in the post-correct and post-error conditions, and the median RT for both FAs and hit responses in the pre-error and post-error conditions for each subject. Speed and accuracy values were used for calculating PIA and PES. As suggested by^39^, the PES was calculated as the RT difference between pre-error and post-error hits. Statistical analyses on behavioral data were performed by means of a *t*-test.

We also obtained signal detection measures of sensitivity (d′) and response criterion (C), calculated as in the equations 1 and 2 respectively:


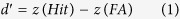



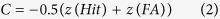


When the hit rate is 1 (i.e., 100% correct,) d′ and C have an unlimited numeric value and cannot be included in the analysis. In order to correct hit rates we adopted the method suggested by^40^, that is to convert the proportions of one to 1-1/(2N), where N is the number of Go trials.

### Electrophysiological recording and analysis

The EEG signal was recorded using the BrainVision^TM^ system (BrainProducts GmbH, Munich, Germany) with 64 electrodes mounted according to the 10-10 International system. All electrodes were referenced to the left mastoid, while the right mastoid served as ground. It should be noted that in the present paradigm the non-symmetric reference produced the same data as the symmetric one. Horizontal and vertical electrooculogram (EOG) were also recorded using electrodes at the right external canthi and below the left eye respectively. Electrode impedances were kept below 5 KΩ. The EEG was digitized at 250 Hz, amplified (band-pass of 0.01–80 Hz including a 50 Hz notch filter) and stored for offline averaging. Artifact rejection was performed prior to signal averaging to discard epochs contaminated by blinks, eye movements or other signals exceeding the amplitude threshold of ±120 μV.

Each condition was averaged in a 2000 ms epoch (from 1100 ms before to 900 ms after the stimulus onset, considered as time 0). To further reduce high frequency noise, the averaged signals were low pass filtered (i.e. Butterworth) at 25 Hz (slope 24 dB/octave). The baseline was defined as the mean voltage during the initial 200 ms of the averaged epochs. Even though this interval may overlap with late activity from some of previous trials, the 1000 ms jitter of the used ISI drastically reduces the activity related to preceding trials, minimizing any effects of this choice. Further, the effect of the baseline used in the present paradigm has been investigated in a recent study^41^ showing that the 1000–2000 ms ISI radically reduces all the activity that are not time related to the considered event.

**Figure 2:**
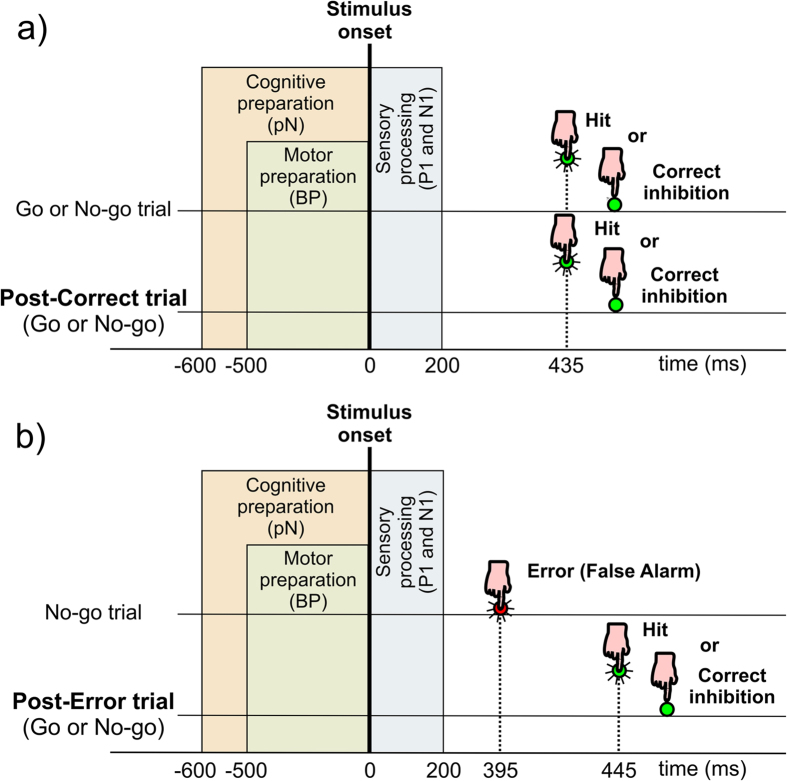
Schematic representations of events in case of (**a**) post-correct and (**b**) post-error trials. In both (**a,b**), the horizontal lines represent two different single trials; the temporal sequence of trials is from the upper line to the lower line. The rectangular areas represent the main brain processes (insensitive to Go/No-go category) taking place as a function of time (not scaled). In the preparation phase, we investigated both the cognitive and motor preparation (as reflected by the BP and pN components respectively); in the post-stimulus phase, we investigated the visual sensory processing (the P1 and N1 components). The figure also shows the mean RTs for post-correct and post-error trials (hits in a and b), and for false alarms (in b).

Since we were mainly interested in the preparatory brain activities and we have already demonstrated the lack of ERPs differences between Go and No-go stimuli, specifically in the cognitive and motor preparation phase (i.e., the pN and the BP components) and in the early sensory response (the P1 and the N1 components)^30^,^32^,^42^, the artifact-free signals were separately segmented into two trial conditions as sketched in Fig. 2: post-correct (i.e., average of Go and No-go following correctly inhibited or responded trials) and post-error (i.e., average of Go and No-go trials following FAs). Because of the averaging of Go/No-go trials, only the pN, the BP, the P1 and N1 components were considered for further analysis. The N2 and P3 components were excluded from analyses because of their sensitivity to Go/No-go categories [e.g. refs 43 and 44]. Similarly, the anterior-distributed prefrontal P2 (pP2, emerging at 300 ms after the stimulus) was not calculated because it is typically larger in Go than No-go trials, reflecting the categorization process^30^,^34^,^41^,^45^.

The pre-stimulus mean amplitudes of each condition were initially compared with a sample-by-sample *t*-test in the prefrontal (Fp1, Fp2) and central (C1, Cz, C2) electrodes previously associated with the pN and BP components: using this method we identified the time windows where the differences were consistently significant. Based on this preliminary analysis, we selected the Fp1 and Fp2 sites in the −600/0 ms time window, and the C1, Cz and C2 sites in the −500/0 ms: the mean amplitude on the selected electrodes was submitted to a repeated measures ANOVA with Site (Fp1, Fp2; C1, Cz, C2) and Condition (post-correct vs. post-error) as factors. The visual evoked P1 and N1 components were respectively measured on the PO8 and PO7 sites as electrodes of maximum activity; for both components, the peak amplitude and latency were submitted to a repeated measures ANOVA with Conditions (post-correct vs. post-error) as repeated measure. Post-hoc comparisons were conducted using the Bonferroni test. The correlation coefficients (Pearson’s r coefficients) were performed in the post-error condition between electrophysiological data and RT of hit responses (no analyses were possible on the RT and percentage of FAs because of their low number in the post-error condition). The overall alpha level was fixed at 0.05.

Note that the relevant comparison in the present study is between post-correct and post-error conditions; thus, our statistical analyses considered these two conditions. The waveforms of error trials are presented in the figures only as control for the possible confounding influence of the error-related activities (see section 3.2.1).

## Results

### Behavioral performance

**Figure 3:**
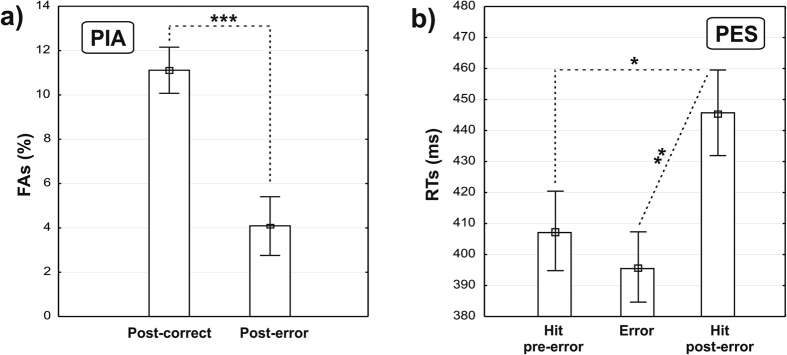
Comparison of behavioral data: (**a**) percentage of false alarms and (**b**) mean response times. PIA: Post-error improvement in accuracy. PES: Post-error slowing. The asterisks denote the p values: *p < 0.05; **p < 0.01; ***p < 0.001.

Statistical analyses on accuracy performance (Fig. 3a) showed that the percentage of FAs decreased significantly from the post-correct (mean = 11.1%, SD = 6.2) to the post-error (mean = 4.04%, SD = 5.36) (t = 5.15, p < 0.0001) condition. After committing an error, 50% of the subjects did not commit a second FA, while 33% committed a second FA, and 17% more than two at least once. Considering the overall low rate of FAs in the post-error condition (4%), two consecutive errors were present in less than 0.5% of trials following FAs.

Analyses on speed performance (Fig. 3b) showed that the post-error RTs (mean = 445 ms, SD = 82.8) were slower than the pre-error RTs (mean = 408 ms, SD = 66.2) (t = −2.1, p < 0.05). The RTs of FAs (mean = 395, SD = 65) were similar to the pre-error RTs (t = −0.83, p > 0.05), and faster than the post-error RTs (t = −2.84, p < 0.01).

Overall, analyses on post-error behavioral data confirmed that error commission led to a more “conservative” performance, characterized by both PIA and PES.

Signal detection analysis revealed a mean d′ of 3.8 (SD = 0.76) and a mean C of −0.61 (SD = 0.23).

### Electrophysiological activities

**Figure 4:**
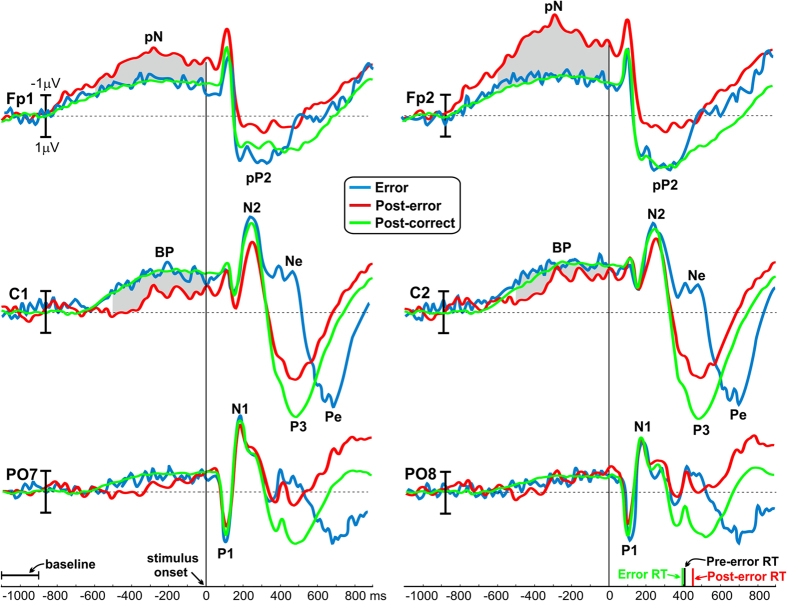
Grand-averaged waveforms of the error (blue), post-error (red) and post-correct (green) trials in bilateral prefrontal (Fp1, Fp2), central (C1, C2) and parieto-occipital (PO7, PO8) sites. Time 0 corresponds to the stimulus onset. The gray areas indicate the time windows considered for statistical analyses. The bars on the right abscissa represent the RTs of different trials.

Figure 4 shows the stimulus-locked activity for post-error (red lines) and post-correct (green lines) conditions over left and right sites on the prefrontal (Fp1, Fp2), central (C1, C2) and parietal-occipital (PO7, PO8) areas. ERPs locked to the erroneous trials are also shown in the figure (blue lines). To facilitate the visual inspection, the entire signal segmentation is reported in the figure even if, as previously indicated, no analyses were performed on the late ERP components like the pP, N2 and P3. Before the stimulus onset, two main components were clearly detectable: the slow-rising pN and the BP components reflecting the cognitive and motor preparation at the level of prefrontal and premotor brain areas respectively. Both the pN and BP of post-correct and error trials were comparable. In contrast, the post-error trials showed specific modulations in the preparatory phase: the pN amplitude was enhanced, especially on the right hemisphere, while the BP was reduced in both hemispheres, with a stronger effect on the left side. After the stimulus onset, the last component (i.e., the Pe) peaked in the error trials at around 700 ms, reaching the baseline value within 900 ms (see C1 and C2). The visual inspection does not suggest significant effects on the P1 and N1 visual components that had similar amplitudes and latencies across conditions.

**Figure 5:**
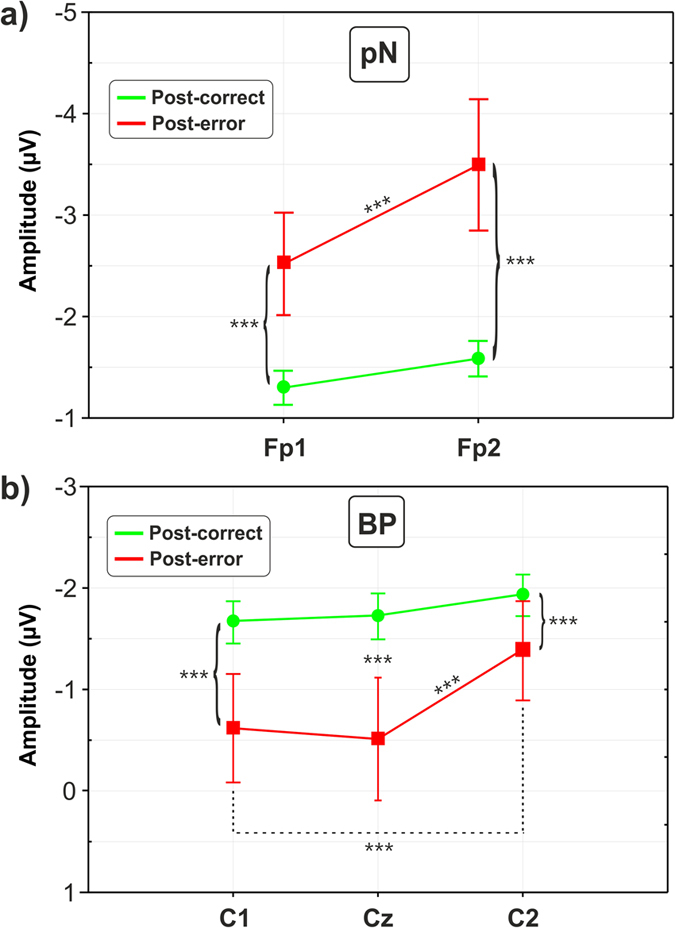
Statistical comparison of the (**a**) prefrontal negativity (pN) and (**b**) Bereitschaftspotential (BP) between post-correct and post-error trials. Vertical bars indicate standard deviations (SD). ***p < 0.001.

ANOVAs on the pre-stimulus ERPs confirmed what was suggested by a visual inspection of Fig. 4. The analyses of the pN component showed a significant effect of Condition (F_1,35_ = 9.1, p < 0.01), revealing more than 100% amplitude enhancement in the post-error (mean = −3 μV, SD = 3.4) with respect to the post-correct (mean = −1.4 μV, SD = 1) condition; further, the effect of Site was significant (F_1,35_ = 12.4, p < 0.01) indicating a larger activity on the Fp2 (mean = −2.5 μV, SD = 2.9) than the Fp1 (mean = −1.9 μV, SD = 2.3) site. The interaction between Condition and Site was also significant (F_1,35_ = 5.3, p < 0.05). *Post-hoc* tests revealed that the laterality effect was mainly accounted by the post-error condition; in fact, the Fp2 activity was larger than Fp1 in the post-error (p < 0.001), and not in the post-correct condition (p > 0.05), as shown in Fig. 5a.

**Figure 6:**
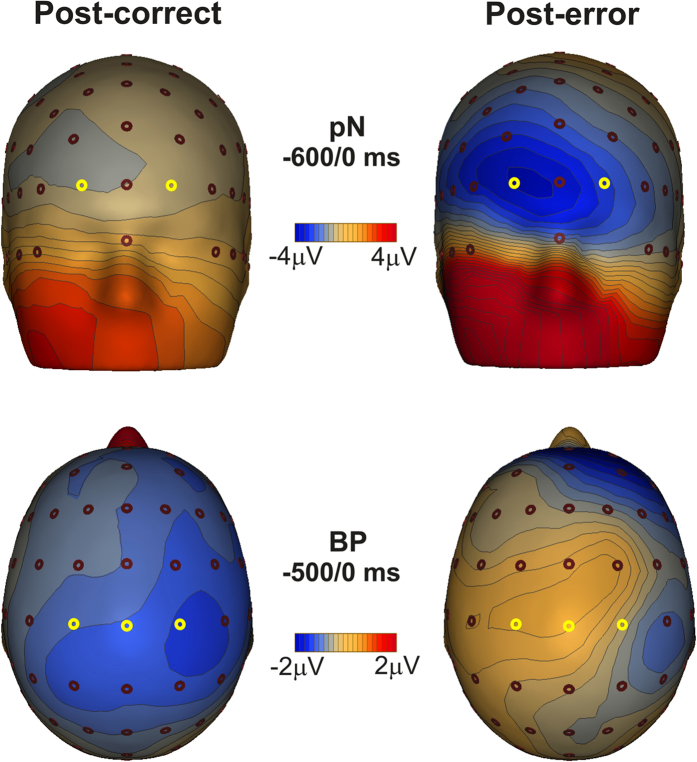
Scalp topographies of the grand-average activities for post-correct and post-error trials. The pN and the BP components are presented on the top and the bottom respectively. The yellow circles indicate the electrodes selected for statistical analyses.

Statistical analyses on the BP component also revealed significant effects of Condition (F_1,35_ = 4.3, p < 0.05) indicating a 56% amplitude reduction of the post-error condition amplitude (mean = −0.8 μV, SD = 3.2) with respect to the post-correct condition amplitude (mean = −1.8 μV, SD = 1.2), and Site (F_2,70_ = 24, p < 0.0001) indicating smaller amplitudes on the left side than the right. The interaction between Condition and Site was significant (F_2,70_ = 11.4, p < 0.0001). *Post-hoc* tests indicated that the BP laterality effect was present in the post-error condition only, where the amplitudes at C1 (mean = −0.6 μV, SD = 3.2) and at Cz (mean = −0.51 μV, SD = 3.6) were smaller than that at the C2 (mean = −1.4 μV, SD = 2.9) site (p < 0.0001 for both comparisons); no laterality effects emerged in the post-correct condition (all ps > 0.05) as shown in Fig. 5b. The topographic distributions of the pre-stimulus brain activities are reported in Fig. 6, showing the surface voltage distribution of the pN and BP components in the two conditions.

No significant effects emerged from the analyses on the amplitude and latency of the visual P1 and N1 components (all ps > 0.05).

A significant correlation was found between the Go RTs and the BP amplitude in the post-error condition: the slower the RTs (i.e. greater the post-error slowing), the smaller the BP at the C1 (r = 0.47, p < 0.01), Cz (r = 0.43, p < 0.01) and C2 (r = 0.47, p < 0.01) sites. In contrast, no significant correlations emerged between RTs and pN amplitudes, neither for the Fp1 (r = 0.02, p > 0.05) nor the Fp2 (r = 0.07, p > 0.05) sites.

Overall, the comparison between post-error and post-correct trials indicates that error commission affects the preparation stage of the subsequent trial. Further, these neural adjustments reflect a genuine adaptive preparatory mechanism being temporally separated from the preceding Ne/Pe complex. Below, additional control analyses are presented to support this point.

### Additional control analyses

**Figure 7:**
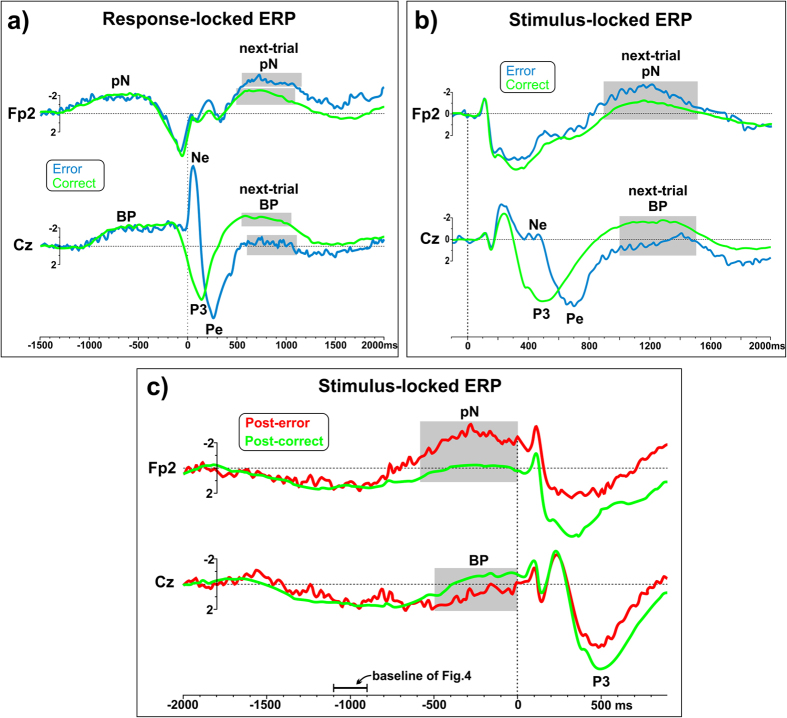
(**a**) Response-locked ERPs of error and correct trials. In the error trials, the Ne and Pe potentials (labeled at Cz) peak largely before the time-window considered for analysis of the next-trial BP. The two waveforms on Fp2 are aligned up to about 300 ms after the motor response. (**b**) Stimulus-locked ERPs of error and correct trials. As in the R-locked segmentation, the error-related activities (Ne and Pe) emerge at Cz well before the time window we adopted to analyze the next-trial BP. The waveforms on the Fp2 site are aligned until about 800 ms after the stimulus onset. (**c**) Stimulus-locked ERPs of post-correct and post-error trials. The epoch starts 2 s before the stimulus onset. The waveforms of the two trials are very similar, up to −800 ms, as in the original analysis of Fig. 4, whose baseline is indicated by a segment.

To support the view that post-error neural adjustments are not confounded with the Ne/Pe complex evoked by error commission, additional controls are reported in Fig. 7, where the pN and BP components are shown on the Fp2 and Cz sites respectively.

Figure 7a,b show the response- and stimulus-locked grand averages respectively. With respect to Fig. 4, these segments adopt a much larger time window and refer to the trials preceding those considered so far (e.g., the error trials instead of the post-error ones). Accordingly, the “next-trial BP” and “next-trial pN” gray areas of Fig. 7 correspond to the BP and pN gray areas on the left side of Fig. 4. For the ERPs of Fig. 7a, the −1500/−1300 ms interval was taken as a baseline. The error-related potentials emerge at Cz for error trials (blue line), and the pN and BP of the next trials are well detectable on the right side of the figure. Despite the response-locked segmentation, the two conditions show the same trend as reported in the previous results section, that is larger pN and smaller BP in the post-error than in the post-correct trials. Note that the different temporal location of the gray areas between conditions depends on the temporal difference between error- and correct-RTs.

Figure 7b shows the same data of Fig. 7a, but the signal is stimulus-locked and a “traditional” baseline is adopted (100 ms pre-stimulus). Again, the pN and BP components of the next trials show the same difference between conditions as reported in the previous results section.

The additional evidence of Fig. 7a,b strengthen the findings of the present study. However, one could argue that, given the large ISI variability (from 1000 to 2000 ms; mean = 1500 ms, SD = 289), the BP and pN components of the next trials were smeared by the 1000 ms time-jitter of the next stimulus. For this reason, an additional control is provided in Fig. 7c. In this latter analysis, we locked the signal to the same trials of Fig. 4, but adopting a larger time window: the epoch was expanded until 2000 ms before the onset of the post-error (and post-correct) stimulus. In this way, the onset of the previous stimuli and the corresponding evoked activities were included in the time window. The main result of Fig. 7c is that the waveforms are nearly aligned up to about −800 ms: hence, the original baseline of Fig. 4 (marked by a segment in both Figs 4 and 7c) did not change the pN and BP difference between trials, which is the focus of the present study. Moreover, this analysis confirms that the large time-jitter of the present paradigm does not allow the Ne and Pe components to emerge in the preparatory phase when ERPs are locked to the post-error stimulus.

## Discussion

The present study confirmed previous behavioral findings showing post-error slowing (PES) and post-error improvement in accuracy (PIA) after error commission. In fact, the trials following errors were characterized by more accurate (i.e., most of the subjects did not commit two consecutive errors) and slower (about 50 ms) responses. However, the main novelty of this study concerns the ERP findings, revealing a reduction of the BP component (especially on the left side) and a bilateral (but more pronounced on the right side) enhancement of the pN component in post-error trials. These results are consistent with both the cognitive^12^ and inhibitory^16^ accounts of the PES. In previous studies that used the same task as the present one^32^,^33^, the pN enhancement has been associated with increased top-down control; consistently, we interpret the pN increase in post-error trials as evidence of increased top-down control during such trials. The cognitive account of the PES is also supported by the reduced BP amplitude in post-error trials, reflecting neural adjustments at premotor level. Based on fMRI^14^,^46^ and EEG^29^,^47^ studies, the BP reduction can be discussed in terms of reduced motor baseline, which in turn is responsible for the slower RTs. At neurophysiological level, the SMA activation (corresponding to the BP enhancement) overcomes the tonic inhibition provided by the output nuclei of the thalamus^48^, while the SMA hypo-activation (marked by the BP reduction) may be functionally interpreted as a mechanism slowing-down the motor response^30^. This view is also supported by the significant correlation between the BP amplitude and the RTs: the smaller the BP, the slower the RTs (corresponding to greater PES). Taking the absence of significant correlations between pN amplitude and RTs into account as well, we can conclude that the speed performance is mostly determined at the SMA level, while the pN component reflects a more indirect, attentional-mediated task control^33^.

It is also interesting to note that the BP reduction in post-error trials was larger over the left hemisphere, further suggesting task-related adjustments for the right responding hand. Conversely, the pN increase was more pronounced on the right hemisphere. This latter laterality effect, together with the observation of the iFg as the source of the pN^34^ and the role of the right-iFg as inhibitory control area^17^,^35^,^49^, underlines the contribution of the inhibitory processes in post-error adjustments, as postulated by the inhibitory account^9^.

Since in our task errors represented infrequent events, current results might also be interpreted in terms of orienting account^18^; in other words, errors might be considered as orienting cues^49^ increasing the inhibitory and attentional control, and reducing the preparatory activity of motor areas.

Overall, because of the simultaneous presence of different neurocognitive processes (top-down, inhibitory and orienting mechanisms), we agree with other authors^11^,^15^,^50^ suggesting that different preparatory processes account simultaneously for the post-error behavioral adjustments. Consistently, post-error neural adjustments were modulated through prefrontal and premotor areas.

It is noteworthy that, differently from studies that used error-prone paradigms by modulating the frequency of target stimuli^7^,^19^, we adopted an equiprobable Go/No-go task. This choice has several advantages, like: maximizing the stimuli uncertainty, minimizing the differences in response conflict between categories^51^, and excluding that frequency-related processes (such as the “oddball” effect), rather than post-error adjustments, account for the ERP modulations.

As also suggested by^19^, the distinction between activities following error is not trivial, because they subtend different neurocognitive processes. Specifically, there are activities related to error-detection and -awareness (the Ne and Pe components respectively^7^,^26^), and others related to the prevention of a second error, as reflected by the reduced premotor activity and the increased top-down control (the BP and pN components in the present study). The temporal segregation of these processes is supported by the present results, showing that the late Pe peaked 700 ms after the stimulus onset, which corresponds to 200/300 ms before the beginning of the preparation phase for the next trial. It is also noteworthy that these processes might well represent a connected cascade of events triggered by error commission. Specifically, after committing an error, the action monitoring system of the medial PFC (as reflected by the ERN/Ne^52^) may act as an alarm facilitating the conscious error perception (the Pe^7^) and recruiting the attentional and inhibitory control networks of the PFC^35^,^53^, as respectively indexed by the bilateral and right-side enhancement of the pN component. Finally, the activation of the MFC might predict the reduced activity of the motor system^15^, as reflected by the reduced BP which in turn is associated with the response slowdown.

On the other hand, the visual sensory processing did not present modulations as effect of the error commission on the previous trial. This result is in line with the findings of our previous studies, showing that the modulation of the P1 and N1 potentials does not reflect inter-trials, state-dependent performance fluctuations, but rather components marking the individual behavioral tendency in performing the whole task^30^,^37^,^45^. In addition, the lack of significant effects at sensory level further confirms the view of the post-error adjustments as brain mechanisms mediated by high-level information processing.

In conclusion, the distinction between error-detection activities and post-error adjustments might be relevant in clinical research as well. In fact, present findings foster the possibility of investigating the neurophysiological correlates of disorders impairing only specific stages of the error-triggered processing ranging from error detection to error prevention, as in the case, for example, of drug addiction^28^, frontal lesion^54^,^55^,^56^ and obsessive-compulsive disorders^57^.

## Additional Information

**How to cite this article**: Perri, R. L. *et al*. How the brain prevents a second error in a perceptual decision-making task. *Sci. Rep.* **6**, 32058; doi: 10.1038/srep32058 (2016).
